# Total and Subtypes of Dietary Fat Intake and Its Association with Components of the Metabolic Syndrome in a Mediterranean Population at High Cardiovascular Risk

**DOI:** 10.3390/nu11071493

**Published:** 2019-06-29

**Authors:** Alicia Julibert, Maria del Mar Bibiloni, Cristina Bouzas, Miguel Ángel Martínez-González, Jordi Salas-Salvadó, Dolores Corella, Maria Dolors Zomeño, Dora Romaguera, Jesús Vioque, Ángel M. Alonso-Gómez, Julia Wärnberg, J. Alfredo Martínez, Luís Serra-Majem, Ramon Estruch, Francisco J. Tinahones, José Lapetra, Xavier Pintó, José Lopez-Miranda, Laura García-Molina, José Juan Gaforio, Pilar Matía-Martín, Lidia Daimiel, Vicente Martín-Sánchez, Josep Vidal, Clotilde Vázquez, Emili Ros, Estefanía Toledo, Nerea Becerra-Tomás, Olga Pórtoles, Karla A. Pérez-Vega, Miquel Fiol, Laura Torres-Collado, Lucas Tojal-Sierra, Rosa Carabaño-Moral, Itziar Abete, Almudena Sanchez-Villegas, Rosa Casas, María Rosa Bernal-López, José Manuel Santos-Lozano, Ana Galera, Lucía Ugarriza, Miguel Ruiz-Canela, Nancy Babio, Oscar Coltell, Helmut Schröder, Jadwiga Konieczna, Domingo Orozco-Beltrán, Carolina Sorto-Sánchez, Sonia Eguaras, Laura Barrubés, Montserrat Fitó, Josep A. Tur, PREDIMED-PLUS Investigators

**Affiliations:** 1 CIBER Fisiopatología de la Obesidad y Nutrición (CIBEROBN), Instituto de Salud Carlos III (ISCIII), 28029 Madrid, Spain; 2 Research Group on Community Nutrition & Oxidative Stress, University of Balearic Islands, 07122 Palma de Mallorca, Spain; 3 Health Research Institute of the Balearic Islands (IdISBa), 07120 Palma de Mallorca, Spain; 4 Department of Preventive Medicine and Public Health, IdISNA, University of Navarra, 31008 Pamplona, Spain; 5 Department of Nutrition, Harvard T. H. Chan School of Public Health, Boston, MA 02115, USA; 6 Human Nutrition Unit, Biochemistry and Biotechnology Department, IISPV, Universitat Rovira i Virgili, 43201 Reus, Spain; 7 Department of Preventive Medicine, University of Valencia, 46100 Valencia, Spain; 8 Unit of Cardiovascular Risk and Nutrition, Institut Hospital del Mar de Investigaciones Médicas Municipal d’Investigació Mèdica (IMIM), 08003 Barcelona, Spain; 9 Miguel Hernández University, ISABIAL-FISABIO, 46020 Alicante, Spain; 10 CIBER Epidemiología y Salud Pública (CIBERESP), Instituto de Salud Carlos III (ISCIII), 28029 Madrid, Spain; 11 Department of Cardiology, OSI ARABA, University Hospital Araba, University of the Basque Country UPV/EHU, 01009 Vitoria-Gasteiz, Spain; 12 Department of Nursing, School of Health Sciences, University of Málaga-IBIMA, 29071 Málaga, Spain; 13 Department of Nutrition, Food Sciences, and Physiology, Center for Nutrition Research, University of Navarra, 31008 Pamplona, Spain; 14 Cardiometabolics Nutrition Group, IMDEA Food, CEI UAM + CSIC, 28049 Madrid, Spain; 15 Institute for Biomedical Research, University of Las Palmas de Gran Canaria, 35016 Las Palmas de Gran Canaria, Spain; 16 Department of Internal Medicine, IDIBAPS, Hospital Clinic, University of Barcelona, 08036 Barcelona, Spain; 17 Virgen de la Victoria Hospital, Department of Endocrinology, University of Málaga, 29010 Málaga, Spain; 18 Department of Family Medicine, Research Unit, Distrito Sanitario Atención Primaria Sevilla, 41013 Sevilla, Spain; 19 Lipids and Vascular Risk Unit, Internal Medicine, Hospital Universitario de Bellvitge, Hospitalet de Llobregat, 08907 Barcelona, Spain; 20 Lipids and Atherosclerosis Unit, Department of Internal Medicine, Maimonides Biomedical Research Institute of Cordoba (IMIBIC), Reina Sofia University Hospital, University of Cordoba, 14004 Cordoba, Spain; 21 Department of Preventive Medicine, University of Granada, 18071 Granada, Spain; 22 Department of Health Sciences, University of Jaen, 23071 Jaen, Spain; 23 Department of Endocrinology and Nutrition, Instituto de Investigación Sanitaria Hospital Clínico San Carlos (IdISSC), 28040 Madrid, Spain; 24 Nutritional Genomics and Epigenomics Group, IMDEA Food, CEI UAM + CSIC, 28049 Madrid, Spain; 25 CIBER Diabetes y Enfermedades Metabólicas (CIBERDEM), Instituto de Salud Carlos III (ISCIII), 28029 Madrid, Spain; 26 Institute of Biomedicine (IBIOMED), University of León, 24071 León, Spain; 27 Department of Endocrinology, IDIBAPS, Hospital Clinic, University of Barcelona, 08036 Barcelona, Spain; 28 Department of Endocrinology, Fundación Jiménez-Díaz, 28040 Madrid, Spain; 29 Lipid Clinic, Department of Endocrinology and Nutrition, Institut d’Investigacions Biomèdiques August Pi Sunyer (IDIBAPS), Hospital Clínic, 08036 Barcelona, Spain; 30 Unidad de Gestión Clínica de Arroyo de la Miel. Distrito de Atención Primaria Costa del Sol, Servicio Andaluz de Salud, 29630 Benalmádena, Spain; 31 Department of Computer Languages and Systems. Universitat Jaume I, 12071 Castellon, Spain

**Keywords:** fatty acids, dietary fat, fat intake, Mediterranean diet, cardiovascular disease risk

## Abstract

**Background:** The effect of dietary fat intake on the metabolic syndrome (MetS) and in turn on cardiovascular disease (CVD) remains unclear in individuals at high CVD risk. **Objective:** To assess the association between fat intake and MetS components in an adult Mediterranean population at high CVD risk. **Design:** Baseline assessment of nutritional adequacy in participants (*n* = 6560, men and women, 55–75 years old, with overweight/obesity and MetS) in the PREvención con DIeta MEDiterránea (PREDIMED)-Plus randomized trial. **Methods:** Assessment of fat intake (total fat, monounsatured fatty acids: MUFA, polyunsaturated fatty acids: PUFA, saturated fatty acids: SFA, trans-fatty acids: trans-FA, linoleic acid, α-linolenic acid, and ω-3 FA) using a validated food frequency questionnaire, and diet quality using 17-item Mediterranean dietary questionnaire and fat quality index (FQI). **Results:** Participants in the highest quintile of total dietary fat intake showed lower intake of energy, carbohydrates, protein and fiber, but higher intake of PUFA, MUFA, SFA, TFA, LA, ALA and ω-3 FA. Differences in MetS components were found according to fat intake. Odds (5th vs. 1st quintile): hyperglycemia: 1.3–1.6 times higher for total fat, MUFA, SFA and ω-3 FA intake; low high-density lipoprotein cholesterol (HDL-c): 1.2 higher for LA; hypertriglyceridemia: 0.7 lower for SFA and ω-3 FA intake. **Conclusions:** Dietary fats played different role on MetS components of high CVD risk patients. Dietary fat intake was associated with higher risk of hyperglycemia.

## 1. Introduction

Obesity and the ensuing metabolic syndrome (MetS) are becoming an epidemic. If recent secular trends continue unabated, up to 20% of the world’s adult population (1.2 billion individuals) is expected to be obese by 2030. The prevalence of type 2 diabetes mellitus (T2DM) and cardiovascular disease (CVD) are also expected to increase by 54% and 22%, respectively [1,2,3,4].

In an effort to tackle the problem of obesity, and in turn cardiovascular risk, nutritional guidelines recommended a global limit on total fat intake, inevitably resulting in an increased intake of simple carbohydrates and decreased intake of healthy unsaturated fatty acids (UFA) [5]. Dietary UFA may prevent the development of metabolic diseases such as T2DM, and reduce cardiovascular events [6]. Moreover, the presumed relationship between dietary saturated fatty acids (SFA) and an increased risk of coronary heart disease (CHD) or CVD may depend on the complexity of these fatty acids and the food matrix in which they are present [7].

Evidence currently available resulting from the dietary fat interventions does not support the current dietary fat guidelines [8]. In 2015, the Dietary Guidelines Advisory Committee emphasized the importance of healthful, food-based diet patterns, revisiting the role of fat in health [9].

The Mediterranean diet (MedDiet) is characterized by high intakes of plant foods (fruits, vegetables, legumes, nuts, and whole grains) and olive oil as the principal source of dietary lipids [10]. This dietary pattern seems to ameliorate metabolic risk factors defining the MetS [11] (and reduce the incidence of cardiovascular events, breast cancer, and T2DM compared with any other diet [12,13].

The PREvención con DIeta MEDiterránea (PREDIMED)-Plus study provides a unique opportunity to assess the association between fat intake and MetS components in an adult Mediterranean population at high CVD risk.

## 2. Methods

### 2.1. Study Design

This research represents a cross-sectional study on baseline data of the PREDIMED-Plus trial. PREDIMED-Plus study is an ongoing 6-year multicenter, parallel-group, randomized trial conducted in 23 Spanish recruiting centers to evaluate the effect of an intensive weight loss program based on an energy-restricted traditional Mediterranean diet (erMedDiet), physical activity promotion, and behavioral support on hard cardiovascular events, in comparison with an usual care intervention only with energy-unrestricted MedDiet (control group) and any advice to increase physical activity. The PREDIMED-Plus study protocol is fully described in a publication by Martínez-González et al. [14] and available at http://predimedplus.com/. The trial was registered in 2014 at the International Standard Randomized Controlled Trial (ISRCT; http://www.isrctn.com/ISRCTN89898870) with number 89898870.

### 2.2. Participants, Recruitment, and Randomization

Eligible participants were community-dwelling adults men aged between 55 and 75 years and women between 60 and 75 years, without documented history of CVD at enrollment, who were overweight or obese (body mass index [BMI] ≥27 and <40 kg/m^2^) and meeting at least 3 criteria for the MetS according to the updated harmonized definition of the International Diabetes Federation and the American Heart Association and National Heart, Lung and Blood Institute [15]: Abdominal obesity for European individuals (WC ≥88 cm in women and ≥102 cm in men), hypertriglyceridemia (≥150 g/dL) or drug treatment for high plasma triglycerides (TG) concentrations, low high-density lipoprotein cholesterol (HDL-cholesterol; ≤50 mg/dL in women and ≤40 mg/dL in men), high blood pressure (systolic blood pressure ≥130 mmHg or diastolic blood pressure ≥85 mmHg or antihypertensive drug treatment), or high fasting plasma glucose (≥100 mg/dL) or drug treatment for T2DM.

From 5 September 2013 to 31 October 2016, a total of 6874 participants were recruited in 23 Spanish centers (universities, hospitals, and research institutes).

**Figure 1:**
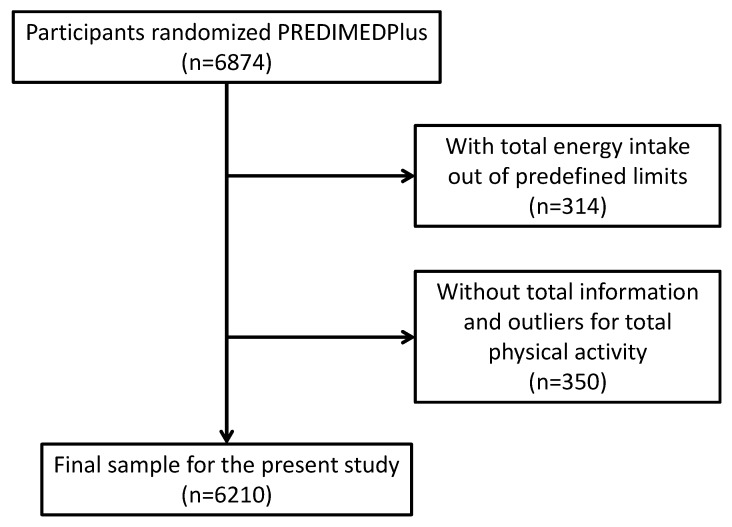
Flow-chart of participants.

The present analysis included 6560 subjects (3387 men and 3173 women (Figure 1) were included). We excluded those participants (*n* = 314) recording extreme total energy intakes (<500 or >3500 kcal/day in women or <800 or >4000 kcal/day in men) [16]. We also excluded participants who did not respond to all the physical activity questionnaires (*n* = 14) and participants reporting outliers for total physical activity expressed as metabolic equivalents of task [METs·min/week (at 3 or more standard deviations [SD] from the mean for each sex)].

### 2.3. Ethics

All participants provided written informed consent, and the study protocol and procedures were approved according to the ethical standards of the Declaration of Helsinki by all the participating institutions.

### 2.4. Dietary Assessment

Registered dietitians collected data on dietary intake at baseline with a semiquantitative 143-item food frequency questionnaire (FFQ), assessing dietary habits over the previous 12 months, repeatedly validated in Spain [17]. Detailed information about the development, reproducibility, and validity of FFQ in the PREDIMED cohort has been previously reported [17,18,19]. For each item, a typical portion size was included and consumption frequencies were registered in 9 categories that ranged from “never or almost never” to “≥6 times/day”. Energy and nutrient intakes were calculated as frequency multiplied by nutrient composition of specified portion size for each food item, using a computer program based on available information in food composition tables [20,21,22,23]. The selected frequency item was converted to a daily intake. For example, if a response was 5–6 times a week, it was converted to 0.78 servings per day (5.5 week/7 days) [19]. For each FFQ food item, we estimated the average amount of food consumed (grams), the average total energy intake, and the average intake of a set of macro-and micronutrients by computing the mean of the values for the individual foods assigned to that item [24].

We also considered the total nutrient intake, the average intake of micronutrients from dietary supplements declared by participants in the FFQ.

### 2.5. Determination of Fat Intake

Dietary intake of total fat and fatty acids: Monounsaturated (MUFA), polyunsaturated (PUFA), and saturated (SFA), trans-fatty acid (TFA), linoleic acid (LA), α-linolenic acid (ALA), and marine ω-3 fatty acid (ω-3 FA) were estimated.

On the other hand, the fat quality index (FQI) was calculated as previously described [16]. Briefly, the FQI was calculated using the ratio (MUFA + PUFA)/(SFA + TFA) as a continuous variable.

Participants were also administered a 17-item Mediterranean dietary questionnaire, a modified version of the previously validated questionnaire used in the PREDIMED trial [25]. Compliance with each of the 17 food habits reflecting an erMedDiet was scored with 1 point and 0 points otherwise. Therefore, a score ranging from 0–17 points, with 0 meaning no adherence and 17 meaning maximum adherence, was developed.

### 2.6. Physical Activity

Physical activity was measured using the Rapid Assessment of Physical Activity Questionnaires (RAPA-1 and RAPA-2) [26] and the validated Minnesota-REGICOR (Registre Gironí del Cor) Short Physical Activity questionnaire [27,28,29]. Metabolic Equivalent of tasks (MET) are calculated by multiplying the intensity (showed by the MET-score) and the duration spent on that activity (measured in minutes). The intensity was assigned based on the compendium of physical activity [30]. Detailed information about the development and reproducibility has been reported [31].

### 2.7. Anthropometric and Blood Pressure Measurements

Anthropometric variables were measured by trained personnel according to the PREDIMED-Plus protocol. Weight and height were measured with high-quality electronic calibrated scales and a wall-mounted stadiometer, respectively. BMI was calculated as weight in kilograms divided by the square of height in meters. Waist circumference (WC) was measured halfway between the last rib and the iliac crest by using an anthropometric tape. Blood pressure was measured in triplicate with a validated semi-automatic oscillometer (Omron HEM-705CP) after 5 min of rest in-between measurements while the participant was in a seated position. All anthropometric variables were determined in duplicate, except for blood pressure (in triplicate).

### 2.8. Blood Collection and Analysis

Samples of fasting blood and urine were also collected after an overnight fast at baseline. Biochemical analyses were performed on fasting plasma glucose, total cholesterol, low high-density lipoprotein cholesterol (HDL-c,) and triglyceride (TG) concentrations in local laboratories using standard enzymatic methods.

### 2.9. Other Health Outcomes

At the baseline visit, additional information related to sociodemographic and lifestyle aspects (education level, civil status, smoking habits, alcohol intake, physical activity, individual and family medical history, and current medication use) was collected.

### 2.10. Statistical Analyses

Analyses were performed with the SPSS statistical software package version 25.0 (SPSS Inc., Chicago, IL, USA). We used the PREDIMED-Plus baseline database generated in August 2017. Overall, 8 nutrients were examined (total fat, MUFA, PUFA, SFA, TFA, LA, ALA and ω-3 FA). Following standard procedures, nutrient intakes were energy-adjusted using the residual method [32,33] and then converted into quintiles. Qualitative variables were expressed as percentages and quantitative variables were expressed as means and SD. Pearson’s chi-square tests and analysis of variance (ANOVA) (for categorical and continuous variables, respectively) were used to compare differences across quintile groups. We used the Bonferroni method to test multiple comparisons across quintile groups. Logistic regression analyses with the calculation of corresponding odds ratio (OR) and the 95% confidence interval (95% CI) were also used to assess the association between MetS components and quintiles of dietary fat and fat subtypes. Results were adjusted for sex, age (continuous variable), BMI (continuous variable), energy intake (continuous variable), alcohol intake (continuous variable), adherence to the MedDiet (continuous variables), total physical activity (continuous variable, expressed as MET·min/week), smoking habit (categorized variable: Current, former and never) and education level (categorized variable: Primary, secondary, university, or graduate) to control for potential confounders. Results were considered statistically significant if *p*-value (2 tailed) <0.05.

## 3. Results

The general characteristics of the study population across quintiles of total and several subtypes of dietary fat intake are shown in Table 1. The percentage of energy from total dietary fat of the participants ranged from 30.5 (SD: 2.9) in the lowest to 48.5 (SD: 3.3) in the highest quintile (mean: 39.4%, SD: 6.5). Specifically, women had significantly higher intakes of total fat, PUFA, SFA, LA and ALA, but lower in the highest quintile of TFA intake. BMI was significantly lower in the highest vs. lowest quintile of total PUFA (0.3 ± 0.13 kg/m^2^, *p* < 0.001), LA (0.3 ± 0.13 kg/m^2^, *p* = 0.020) and ALA (0.6 ± 0.13 kg/m^2^, *p* < 0.001). Conversely, BMI was significantly higher in the highest quintiles of total fat and SFA. Mean physical activity (expressed as METs·min/week) was significantly higher in the highest quintile of MUFA, PUFA, ALA and ω-3 FA intakes, but lower in the highest quintile of SFA and TFA intakes. Statistical significant differences in education level were also found between quintile groups of total fat, MUFA, SFA, TFA, LA, and ω-3 FA intake. Statistical significant differences in smoking habits were also found between quintile groups of PUFA, SFA, TFA, ALA, and ω-3 FA.

The nutrient intake and food consumption of the participants as per quintiles of total and several subtypes of dietary fat were also assessed (Table 2 and Table 3). Participants in the highest quintile of total dietary fat intake had significantly lower intakes of energy, carbohydrates, protein, and fiber, but higher intakes of all subtypes of fat (PUFA, MUFA, SFA, TFA, LA, ALA, and ω-3 FA). Participants in the highest quintiles of PUFA, LA, and ALA intake had lower TFA and cholesterol intakes but higher fiber intake (except for ALA quintiles). Fiber intake was also higher in participants in the highest quintile of ω-3 FA intake. Participants in the highest quintile of ω-3 FA and SFA intake had significantly higher intake of protein and cholesterol. Cholesterol intake was also higher in participants in the highest quintile of TFA. FQI increased significantly with increasing quintiles of total and all subtypes of dietary intake except for SFA and TFA.

Consumption of olive oil, nuts, total fish, and total meat increased significantly with increasing quintiles of total dietary fat intake, whereas consumption of fruits, vegetables, legumes, total cereals, dairy products, cookies, and alcohol decreased. Similar results were obtained when MUFA quintiles were assessed. In contrast, participants in the highest quintile of PUFA intake had higher consumption of vegetables and legumes but lower total meat consumption. Participants in the highest quintile of ω-3 FA intake had higher consumption of fruits, vegetables, legumes, and total meat; and highest quintile of ALA intake had higher consumption of vegetables but lower of fruits, legumes, total cereals and olive oil, as well as meat. Otherwise, participants in the highest quintile of SFA intake had lower consumption of fruits, vegetables, legumes, nuts, total fish, total cereals, and dairy products, but higher consumption of total meat and cookies. Highest quintile of TFA intake was also associated with higher consumption of total cereals, dairy products, total meat, cookies and alcohol but lower consumption of fruits and vegetables. Overall, participants in the highest quintile of total and all subtypes of dietary fat intake had a significantly higher MedDiet score, except for SFA and TFA intake.

Prevalence of hyperglycemia was significantly higher in the highest quintiles of total and all subtypes of dietary fat intake except for TFA and ALA intake. Prevalence of low HDL-c was also higher in participants with the highest quintile of total fat, PUFA and LA intake but lower in the highest quintiles of ω-3 FA, MUFA, SFA, and TFA intake; and abdominal obesity prevalence was higher in participants with the highest quintile of ALA intake. Contrarily, the prevalence of hypertriglyceridemia was lower in participants with high ω-3 FA intake. Hypertension prevalence did not differ significantly according to intake of any type of fat.

Multivariate adjusted Odds Ratios (ORs) for components of the MetS across quintiles of total dietary fat intake and several subtypes of dietary fat intake are presented in Table 4. After adjustment for potential confounders (i.e., age, sex, BMI, smoking habit, education, energy, and alcohol intake, adherence to the MedDiet and physical activity), the OR of hyperglycemia were 1.2–1.6 times higher from the fourth-fifth quintile (Q4–Q5) of total fat and MUFA intakes compared with the first quintile; the OR of hyperglycemia were 1.3–1.6 times higher from the third-fifth quintile (Q3–Q5) of SFA intake compared with the first quintile. However, the OR for ω-3 FA intake was 1.3 times higher only for the fifth quintile (Q5) compared with the first quintile. The OR of low HDL-c was also 1.2 times higher for the fifth quintile of LA compared with the first quintile. Contrarily, the OR of hypertriglyceridemia were 0.7–0.8 times lower from the third quintiles (Q3–Q5) of SFA intake and the OR for ω-3 FA intake was 0.7 times lower only for the fifth quintile compared with the first quintiles.

## 4. Discussion

In this cross-sectional study we evaluated the association of total dietary fat and specific subtypes of dietary fat intake with the components of MetS in a Mediterranean population at high cardiovascular risk. The most important finding of the present study is a significant increase in the risk of hyperglycemia among participants in the upper quintiles of total dietary fat, SFA, MUFA, and ω-3 FA intake, a significant increase in the risk of low HDL-c levels among participants in the upper quintile of LA, and a significant decrease in the risk of hypertriglyceridemia among participants in the upper quintiles of SFA and ω-3 FA intakes.

Controversial results in relation to hyperglycemia and dietary fat intake associations have been observed in the literature. Our results are in accordance with those reporting that fat intake is positively associated with the prevalence of impaired fasting glucose [34,35] and lately diagnosed and undiagnosed T2DM [34,36,37]. Nevertheless, several studies did not highlight any association between total fat intake and TD2M risk [38,39,40,41,42]. Therefore, dietary fats could affect insulin resistance or risk of diabetes through several mechanisms. In the state of insulin resistance, lipogenesis is inhibited and lipolysis is exalted in adipocytes, which increases concentrations of circulating fatty acids. Consequently, typical dyslipidaemia is characterized by elevated TG, lowered HDL-C, and small and dense LDL-C particles, established as a risk factor for CVD, and associated with hyperinsulinaemia [43]. In the vascular-metabolic Clinica Universidad de Navarra (VM-CUN) cohort, the prediction ability of triglyceride-glucose index (TyG index) and fasting plasma glucose was compared to predict incident T2DM and reported that its predictive ability is better than that of the Homeostatic Model Assessment for Insulin Resistance (HOMA-IR) [44]. Moreover, the higher level of TyG index was significantly associated with an increased risk of incident T2DM [45] and developing CVD [46].

On the other hand, in our study high SFA intake and hyperglycemia were also positively associated. A recent systematic review also pointed out positive associations between SFA intake and insulin sensitivity in observational studies, but any association between SFA intake and incidence of T2DM in prospective studies [47]. Another systematic review and meta-analysis of observational studies did not found any association between SFA and T2DM [48]. The (Lipids, Genes and Metabolic Syndrome Study (LIPGENE study) showed that MetS subjects responded differently to dietary fat modification according to their homeostasis model assessment-insulin resistance (HOMA-IR) status. Insulin-resistant MetS subjects with the highest HOMA-IR decreased fasting insulin and HOMA-IR concentrations after consumption of a high MUFA (HMUFA) diet and high-complex carbohydrate (LFHCC) diet supplemented with long-chain n-3 PUFA diet, and these decreases in the two markers were significantly lower than with the high SFA (HSFA) diet. Conversely, fasting insulin and HOMA-IR concentrations increased in the least insulin-resistant group after consumption of a high SFA (HSFA) diet [49].

Our results also show a significant increase in the prevalence of hyperglycemia with increasing MUFA and ω-3 FA intake. However, our results could be attributed to lower consumption of fruits, vegetables, legumes, fiber, and a higher meat intake among participants with the highest MUFA intake but contrary to the participants with the lowest ω-3 FA intake. A recent systematic review and meta-analysis of randomized controlled feeding trials showed beneficial effects of MUFA and PUFA on glucose-insulin homeostasis. Replacement of 5% dietary energy from carbohydrates or SFA with 5% dietary energy from either MUFA or PUFA lowered glycosylated hemoglobin A1C (HbA1c) and HOMA-IR. Replacement of 5% dietary energy from carbohydrates, SFA, or MUFA with PUFA also showed beneficial effects on insulin secretion ability [50]. Therefore, the modification of an individual dietary pattern to regularly include foods rich in MUFA and PUFA, such as the MedDiet, can benefit individuals with MetS and hyperglycemia or TD2M [51].

We also found that the OR for hypertriglyceridemia was lower in the upper quintiles of SFA intake (Q3–Q5). Contrarily to our results, a positive association of SFA intake with serum TG has been found in the literature [52,53,54,55]. Otherwise, a previous systematic review pointed out that the replacement of 1% dietary energy from SFA with MUFA or PUFA lowered TG levels [56]. However, our results could be attributed to the synergistic effect of high olive oil consumption (a healthy source of MUFA) among participants with the highest SFA intake. However, other studies have not found significant association between MUFA and MetS components, such as hypertriglyceridemia [52,57].

The present study also showed a significant increase in risk of hypertriglyceridemia in the upper quintile of ω-3 FA intake. Accordingly, some studies have shown that the intake of ω-3 FA, eicosapentaenoic acid (EPA), and docosahexaenoic acid (DHA), be effective in reducing plasma TG concentrations [47,52,58,59]. Other authors have also reported that low fat diets enriched with PUFA or replaced by healthy sources of fats (fish, avocado, nuts, broccoli, thistle, olives, linseed, and canola oil, etc.) or healthy sources of carbohydrates (whole grains, legumes, vegetables, and fruits) also decreased TG levels [49,60,61,62,63,64,65].

A previous systematic review of clinical trials found that some studies have observed that various conjugated linoleic acid (CLA) isomers, administered as supplements or CLA-enriched products, decreases HDL-c [66]. Another systematic review pointed out an inverse association between LA intake and CHD risk [67]. Recently, Yanai et al. [68] pointed out that TFA is significantly associated with reduction of HDL-c and coronary risks, whereas MUFA, plant sterols and stanols intake (except policosanol) may not affect HDL-c. Conversely, fish oils consumption, especially DHA consumption, may be favorably associated with HDL metabolism [68]. In our study, LA intake but not TFA was significantly associated with low HDL-c levels.

It is also noting that no association between TFA intake and components of MetS was observed in our population; perhaps it is due to the intake of this type of fat being low in the elderly Mediterranean population, who consume low amounts of processed food [69]. However, the consumption of TFA has been identified as an important and modifiable risk factor for CHD [70]. Emerging data suggest that TFA is associated with all causes of mortality, total CHD, and CHD mortality, probably because of higher levels of intake of industrial TFA (products of partial hydrogenation of vegetable oils) than ruminant TFA (meats and dairy products of cows, sheep, and goats) [48].

Lately, abdominal obesity and HTN are not significantly associated with dietary fat intake, although these results could be attributed to the high prevalence of both MetS components in our population. However, another study showed a significant rise in the risk of abdominal obesity (OR 1.61, CI 1.23–2.13) and HTN (OR 1.39, CI 1.06–1.81) with increasing fat intake [35]. Moreover, findings from the Food4Me study showed a strong association between fat intake (total fat, MUFA, and SFA) and obesity risk [71], which in agreement with two Cochrane meta-analyses [70,72] comparing the weight loss effects of a low fat diet with usual diet showed an effect size of −1.5 kg and was mirrored by reductions in BMI (−0.5 kg/m^2^) and WC (−0.3 cm). Otherwise, a high-quality, moderately high-fat eating pattern (especially unsaturated fatty acids: PUFA and MUFA) like the MedDiet may have beneficial effects on BW and obesity [73]. A recent meta-analysis also provided evidence that high MUFA diets, as well as the Dietary Approaches to Stop Hypertension (DASH) and Mediterranean diets, and supplementation with ω-3 FA (EPA+DHA) effectively lowers blood pressure [47]. No evidence of harmful effects of reducing SFA intakes on blood pressure has been observed in the literature [70].

Our results may help to highlight the fact that dietary recommendations should focus not on lowering the total fat content of the diet but rather on specific types of fats and carbohydrates and, more importantly, on specific foods and overall dietary patterns [9] for individuals at high CVD risk.

## 5. Strengths and Limitations

Our study also has various strengths. The large study sample is highly representative of Spanish older adults with MetS (*n* = 6560), and the use of a standardized protocol reduces information bias about food intake, socioeconomic and lifestyles variables. Some methodological limitations should be acknowledged. First, the cross-sectional study nature; thus, causal inferences cannot be drawn. Second, the FFQ, the source of information to assess dietary fat intake, could overestimate the intake of certain food groups even having been validated. Third, we excluded participants with energy intake out of predefined ranges, to avoid information bias [15]. Previously, in the PREDIMED study, 827 participants who had extreme values for total energy intake or any micronutrient intake out of the predefined values were also excluded in the nutritional adequacy analysis [15]. Moreover, the present findings cannot be extrapolated to other population groups given that our study participants are senior adults with overweight/obesity and MetS. Although there have been controversies regarding the criteria of MetS, the harmonizing worldwide criteria have been agreed on by international academic societies [15]. However, some professional societies have pointed out the limitations of MetS as clinical and epidemiologic too [74,75,76]. Finally, the overestimation of the prevalence ratios derived from the OR when logistic regression is applied.

## 6. Conclusions

These data suggest a potential different role of types of dietary fat on the MetS components of individuals at high cardiovascular risk. Our main findings suggest that the intake of dietary fat was associated with a higher risk of hyperglycemia. It is likely that the effects of dietary fat intake on cardiometabolic syndrome will be influenced by the combination of nutrients of the food consumed. Therefore, the type of dietary fat should be considered for future dietary recommendations to decrease risk of MetS at a population level.

## Figures and Tables

**Table 1:** Lifestyle characteristics and Metabolic Syndrome components according to total dietary fat and specific types of fat (g/day).

	Total Fat			MUFAs			PUFAs			SFAs		
	Q1	Q5	*p* Value	Q1	Q5	*p* Value	Q1	Q5	*p* Value	Q1	Q5	*p* Value
Participants, *n*	1294	1291		1312	1312		1312	1312		1312	1312	
Age, years	65.1 ± 5.0	64.7 ± 4.9	0.164	65.1 ± 5.0	64.8 ± 4.8	0.088	64.7 ±5.0 *	65.3 ± 4.9 *	0.016	65.2 ± 5.0 *	64.5 ± 5.0 *	<0.001
Women, %	43.4	48.5	<0.001	45.0	48.8	0.094	40.5	49.2	<0.001	42.5	46.7	<0.001
BMI, kg/m^2^	32.4 ± 3.4	32.8 ± 3.5 ^NS^	0.005	32.4 ± 3.4	32.7 ± 3.6	0.226	32.6 ± 3.4 *	32.3 ± 3.4 *	<0.001	32.3 ± 3.3 *	32.8 ±3.5 *	<0.001
Smoking habit, %												
Current	11.6	12.0	0.820	11.1	12.0	0.446	14.1	11.5	0.002	11.2	12.5	0.021
Former	44.0	44.6		42.2	44.9		47.1	41.7		46.7	45.2	
Never	44.5	43.4		46.7	43.1		38.8	46.8		42.1	42.3	
Education, %												
Primary	52.3	42.6	<0.001	52.8	44.5	0.001	46.2	48.7	0.268	50.3	40.3	<0.001
Secondary	28.3	30.3		27.7	29.6		30.6	28.2		27.8	34.7	
University or graduate	19.4	27.1		19.5	25.9		23.2	23.1		21.9	25.0	
Total physical activity, *n* †	1213	1225		1226	1240		1225	1251		1235	1248	
Total physical activity, MET·min/week †	2394 ± 2024	2440 ± 1876	0.912	2396 ± 1965 *	2486 ± 1883 *	0.027	2322 ± 2019	2529 ± 1875 ^NS^	0.025	2628 ± 2042 *	2281 ± 1918 *	<0.001
Males	2796 ± 2268	2632 ± 2029	0.233	2745 ± 2177	2744 ± 2029	0.146	2674 ± 2231	2838 ± 2088	0.366	3053 ± 2256 *	2572 ± 2163 *	0.001
Females	1868 ± 1500 *	2233 ± 1672 *	0.004	1970 ± 1569 *	2213 ± 1673 *	0.005	1799 ± 1513 *	2205 ± 1559 *	<0.001	2039 ± 1518	1950 ± 1531	0.458
MetS components, %												
High blood pressure	93.0	91.7	0.328	92.5	91.3	0.300	92.8	92.0	0.724	92.5	91.3	0.495
Hyperglycaemia	73.9	80.3	<0.001	74.0	80.0	<0.001	73.3	75.6	0.014	73.2	78.3	0.032
Hypertriglyceridemia	58.7	56.6	0.139	57.0	56.0	0.889	56.8	55.2	0.111	57.9	53.4	0.120
Low HDL-cholesterol	38.7	44.4	0.004	40.4	44.7	0.171	40.9	46.3	0.015	40.2	42.1	0.068
Abdominal obesity	95.2	96.7	0.180	95.4	96.6	0.317	95.6	96.6	0.657	95.5	96.4	0.380
Males	92.0	94.3	0.387	92.2	94.0	0.386	93.1	94.2	0.704	92.6	93.8	0.503
Females	99.5	99.4	0.944	99.3	99.4	0.453	99.2	99.2	0.543	99.5	99.3	0.990
	***Trans* FA**			**Linoleic acid**			**Linolenic acid**			**ω-3 FA**		
Participants, *n*	1312	1312		1312	1312		1312	1312		1312	1312	
Age, years	65.8 ± 4.7 *	64.0 ± 5.1 *	<0.001	64.6 ± 5.0 *	65.4 ± 4.9 *	0.001	63.9 ± 5.0 *	65.6 ± 4.8 *	<0.001	65.1 ± 5.0	65.1 ± 4.8	0.345
Women, *n* (%)	60.9	37.6	<0.001	40.2	48.2	<0.001	29.9	56.6	<0.001	47.6	51.2	0.217
BMI, kg/m^2^	32.3 ± 3.4	32.6 ± 3.5	0.074	32.6 ± 3.4	32.3 ± 3.3 ^NS^	0.020	32.7 ± 3.4 *	32.1 ± 3.3 *	<0.001	32.7 ± 3.4	32,5 ± 3.4	0.387
Smoking habit, *n* (%)												
Current	8.9	14.0	<0.001	14.0	12.2	0.007	16.7	9.5	<0.001	14.4	10.3	0.003
Former	39.7	47.0		47.1	41.8		49.6	39.4		42.4	45.1	
Never	51.4	39.0		39.0	46.0		33.8	51.1		43.2	44.6	
Education, *n* (%)												
Primary	57.0	41.3	<0.001	44.1	50.5	0.003	46.6	50.1	0.286	54.2	47.2	<0.001
Secondary	24.6	31.6		30.8	28.1		29.9	27.6		27.4	27.8	
University or graduate	18.4	27.1		25.2	21.4		23.5	22.3		18.3	25.0	
Total physical activity, *n* †	1248	1230		1229	1249		1216	1251		1237	1253	
Total physical activity, MET·min/week †	2572 ± 1924	2373 ± 1977	0.075	2445 ± 2098	2499 ± 1884	0.130	2454 ± 2099 *	2621 ± 1961 *	0.002	2227 ± 1952 *	2673 ± 2038 *	<0.001
Males	3107 ± 2208 *	2580 ± 2168*	<0.001	2801 ± 2332	2852 ± 2095	0.491	2695 ± 2241 *	3080 ± 2230 *	0.015	2657 ± 2168	2983 ± 2341 ^NS^	0.048
Females	2225 ± 1624 *	2027 ± 1550 *	0.006	1908 ± 1539	2114 ± 1534	0.191	1852 ± 1541 *	2262 ± 1636 *	<0.001	1744 ± 1541 *	2372 ± 1639 *	<0.001
MetS components, *n* (%)												
High blood pressure	91.9	92.3	0.108	92.9	92.0	0.313	92.9	91.6	0.566	91.8	91.5	0.068
Hyperglycaemia	76.2	75.2	0.232	73.4	75.2	0.047	74.1	75.8	0.373	74.0	79.0	0.020
Hypertriglyceridemia	55.2	55.6	0.611	55.6	56.4	0.847	57.2	53.2	0.186	59.1	50.8	<0.001
Low HDL-cholesterol	43.5	42.2	0.863	39.9	47.0	<0.001	41.5	44.7	0.586	45.8	41.1	0.147
Abdominal obesity	95.9	95.7	0.360	95.6	96.3	0.850	94.4	97.0	0.009	95.8	97.1	0.179
Males	90.6	93.7	0.170	93.1	93.5	0.948	92.2	94.0	0.414	92.6	94.7	0.338
Females	99.2	99.2	0.587	99.2	99.2	0.479	99.7	99.2	0.343	99.4	99.4	0.210

Abbreviations: BMI, body mass index; FA, fatty acids; HDL-cholesterol, high density lipoprotein cholesterol; MetS, Metabolic Syndrome; MET, metabolic equivalent of task; MUFAs monounsaturated fatty acids; PUFAs, polyunsaturated fatty acids; SFAs, saturated fatty acids. All values are means ± SDs unless otherwise indicated. All quartiles were included in theanalysis. Pearson’s chi-square test was used for categorical variables, and 1-factor ANOVA was used for continuous variables. * *p* < 0.05 for between-group changes, after adjustment for multiple comparisons with the Bonferroni method. ^NS^ No statistical significance after post hoc test. † Participants who not responded the physical activity questionnaires and participants reporting outliers for total physical activity expressed as MET·min/week (at 3 or more standard deviations from the mean) were excluded from the analysis (i.e., 154 men and 196 women).

**Table 2:** Nutrient intake according to total dietary fat and specific types of fat (g/day).

	Total Fat			MUFAs			PUFAs			SFAs		
	Q1	Q5	*p* Value	Q1	Q5	*p* Value	Q1	Q5	*p* Value	Q1	Q5	*p* Value
Participants, *n*	1294	1291		1312	1312		1312	1312		1312	1312	
Energy intake, kcal/day	2446 ± 579 *	2432 ± 509 *	<0.001	2415 ± 594 *	2417 ± 516 *	<0.001	2539 ± 534 *	2498 ± 517 *	<0.001	2532 ± 533 *	2456 ± 551 *	<0.001
Carbohydrate intake, % total energy	48.6 ± 5.4 *	33.3 ± 4.3 *	<0.001	48.0 ± 5.6 *	34.1 ± 4.9 *	<0.001	45.7 ± 6.5 *	37.7 ± 6.2 *	<0.001	46.3 ± 6.3 *	36.0 ± 5.7 *	<0.001
Protein intake, % total energy	16.7 ± 3.0 *	15.9 ± 2.5 *	<0.001	17.0 ± 3.0 *	15.7 ± 2.4 *	<0.001	16.3 ± 2.7 *	16.2 ± 2.7 *	<0.001	15.9± 2.7 *	16.7 ± 2.7 *	<0.001
Fat intake, % total energy	30.5 ± 2.9 *	48.5 ± 3.3 *	<0.001	31.4 ± 3.9 *	47.6 ± 4.1 *	<0.001	33.7 ± 5.1 *	43.7 ±5.9 *	<0.001	33.5 ± 5.1 *	44.8 ± 5.5 *	<0.001
PUFA, % total energy	5.0 ± 1.3 *	7.8 ± 2.0 *	<0.001	5.5 ± 1.9 *	7.4 ± 1.6 *	<0.001	4.3 ± 0.5 *	9.1 ± 1.5 *	<0.001	6.0 ± 1.8 *	6.4 ± 1.8 *	<0.001
MUFA, % total energy	14.8 ± 2.2 *	26.2 ± 3.5 *	<0.001	14.3 ± 1.8 *	26.9 ± 2.9 *	<0.001	17.0 ± 3.2 *	22.2 ± 5.0 *	<0.001	17.5 ± 4.1 *	22.7 ± 4.4 *	<0.001
SFA, % total energy	8.2 ± 1.5 *	11.7 ± 1.9 *	<0.001	8.7 ± 1.8 *	11.0 ± 1.9 *	<0.001	9.6 ± 2.2 *	10.0 ± 1.8 *	<0.001	7.5 ± 0.9 *	12.8 ± 1.4 *	<0.001
*Trans* FA, g/day	0.52 ± 0.3 *	0.71 ± 0.5 *	<0.001	0.57 ± 0.4 *	0.63 ± 0.4 *	<0.001	0.66 ± 0.4 *	0.59 ± 0.4 *	<0.001	0.39 ± 0.3 *	0.94 ± 0.5 *	<0.001
Linoleic acid, g/day	11.1 ± 4.5 *	17.4 ± 6.4 *	<0.001	12.0 ± 5.7 *	16.3 ± 5.4 *	<0.001	9.8 ± 3.1 *	20.8 ± 5.5 *	<0.001	13.7 ± 5.4 *	14.3 ± 5.8 *	<0.001
Linolenic acid, g/day	1.2 ± 0.6 *	1.8 ± 0.8 *	<0.001	1.3 ± 0.7 *	1.7 ± 0.7 *	<0.001	1.1 ± 0.3 *	2.3 ± 0.8 *	<0.001	1.4 ± 0.7 *	1.6 ± 0.6 *	<0.001
ω-3 FA, g/day	1.48 ± 0.9 *	1.65 ± 0.9 *	<0.001	1.52 ± 0.9 *	1.61 ± 0.8 *	0.001	1.39 ± 0.8 *	1.72 ± 0.89 *	<0.001	1.59 ± 0.9	1.55 ± 0.8	0.360
FQI, score	2.4 ± 0.5 *	3.0 ± 0.7 *	<0.001	2.29 ± 0.5 *	3.15 ± 0.7 *	<0.001	2.26 ± 0.5 *	3.16 ± 0.7 *	<0.001	3.12 ± 0.7 *	2.24 ± 0.4 *	<0.001
Cholesterol (mg/day)	365 ± 113 *	399 ± 119 *	<0.001	378 ± 122	383 ± 115	0.182	394 ± 134 *	381 ± 111 *	<0.001	346 ± 106 *	438 ± 125 *	<0.001
Fibre intake (g/day)	29 ± 10.0 *	23.8 ± 7.7 *	<0.001	27.9 ± 9.2 *	25.0 ± 8.4 *	<0.001	26.6 ± 9.0 *	28.3 ± 9.1 *	<0.001	31.2 ± 10.3 *	22.6 ± 7.2 *	<0.001
	***Trans* FA**			**Linoleic acid**			**Linolenic acid**			**ω-3 FA**		
Participants, *n*	1312	1312		1312	1312		1312	1312		1312	1312	
Energy intake, kcal/day	2001 ± 467 *	2739 ± 522 *	<0.001	2541 ± 528 *	2489 ± 518 *	<0.001	2894 ± 419 *	2271 ± 517 *	<0.001	2176 ± 549 *	2517 ± 520 *	<0.001
Carbohydrate intake, % total energy	46.3 ± 6.3 *	36.0 ± 5.7 *	<0.001	44.9 ± 6.7 *	38.0 ± 6.2 *	<0.001	44.4 ± 6.5	38.0 ± 6.4	<0.001	43.0 ± 7.2 *	39.1 ± 6.3 *	<0.001
Protein intake, % total energy	17.1 ± 3.2 *	16.0 ± 2.5 *	<0.001	16.8 ± 3.0 *	15.9 ± 2.5 *	<0.001	15.2 ± 2.2	17.2 ± 2.9	<0.001	15.5 ± 2.8 *	17.8 ± 2.6 *	<0.001
Fat intake, % total energy	38.1 ± 7.2 *	40.9 ± 6.0 *	<0.001	34.0 ± 5.4 *	43.7 ± 5.9 *	<0.001	34.0 ± 5.4	43.7 ± 5.9	<0.001	38.5 ± 7.0 *	40.1 ± 6.2 *	<0.001
PUFA, % total energy	6.4 ± 2.0 *	6.3 ± 1.7 *	0.044	4.4 ± 0.6 *	9.1 ± 1.6 *	<0.001	5.4 ± 1.5	8.5 ± 1.7	<0.001	6.0 ± 2.0 *	6.8 ± 1.8 *	<0.001
MUFA, % total energy	20.6 ± 5.3	20.6 ± 4.2	0.581	17.1 ± 3.3 *	22.2 ± 5.0 *	<0.001	18.2 ± 3.8	21.8 ± 5.0	<0.001	20.1 ± 4.9 *	20.7 ± 4.5 *	0.003
SFA, % total energy	7.5 ± 0.9 *	12.8 ± 1.4 *	<0.001	9.7 ± 2.3 *	10.0 ± 1.8 *	<0.001	9.4 ± 1.8	9.9 ± 2.1	<0.001	9.8 ± 2.1	9.9 ± 1.9	0.168
*Trans* FA, g/day	0.18 ± 0.1 *	1.18 ± 0.4 *	<0.001	0.65 ± 0.4 *	0.62 ± 0.4 *	<0.001	0.70 ± 0.4 *	0.52 ± 0.4 *	<0.001	0.57 ± 0.4 *	0.58 ± 0.4 *	0.015
Linoleic acid, g/day	11.4 ± 5.1 *	16.1 ± 6.1 *	<0.001	9.6 ± 2.9 *	20.9 ± 5.4 *	<0.001	14.2 ± 5.3 *	17.5 ± 5.7 *	<0.001	12.5 ± 5.8 *	14.4 ± 5.6 *	<0.001
Linolenic acid, g/day	1.3 ± 0.7 *	1.7 ± 0.7 *	<0.001	1.1 ± 0.3 *	2.2 ± 0.8 *	<0.001	1.2 ± 0.3 *	2.3 ± 0.8 *	<0.001	1.2 ± 0.7 *	1.7 ± 0.7 *	<0.001
ω-3 FA, g/day	1.54 ± 0.9 *	1.56 ± 0.8 *	0.170	1.62 ± 0.9 *	1.58 ± 0.9 *	0.009	1.56 ± 0.8 *	1.69 ± 0.9 *	<0.001	0.63 ± 0.2 *	2.92 ± 0.4 *	<0.001
FQI, score	3.25 ± 0.7 *	2.29 ± 0.5 *	<0.001	2.25 ± 0.5 *	3.14 ± 0.7 *	<0.001	2.51 ± 0.5 *	3.08 ± 0.7 *	<0.001	2.67 ± 0.7 *	2.78 ± 0.6 *	<0.001
Cholesterol (mg/day)	300 ± 90 *	461 ± 124 *	<0.001	406 ± 135 *	376 ± 111 *	<0.001	427 ± 140 *	361 ± 106 *	<0.001	305 ± 100 *	445 ± 116 *	<0.001
Fibre intake (g/day)	26.2 ± 9.0	25.8 ± 8.4	0.784	27.1 ± 9.1 *	27.6 ± 9.1 *	<0.001	28.1 ± 9.0 *	28.0 ± 9.2 *	<0.001	23.1 ± 8.2 *	28.7 ± 9.1 *	<0.001

Abbreviations: FA, fatty acids; FQI, fat quality index; MUFAs, monounsaturated fatty acids; PUFAs, polyunsaturated fatty acids; SFAs, saturated fatty acids. All values are means ± SDs unless otherwise indicated. All quartiles were included in the analysis. Pearson’s chi-square test was used for categorical variables, and 1-factor ANOVA was used for continuous variables. * *p* < 0.05 for between-group changes, after adjustment for multiple comparisons with the Bonferroni method.

**Table 3:** Food consumption according to total dietary fat and specific types of fat (g/day).

	Total Fat			MUFAs			PUFAs			SFAs		
	Q1	Q5	*p* Value	Q1	Q5	*p* Value	Q1	Q5	*p* Value	Q1	Q5	*p* Value
Participants, *n*	1294	1291		1312	1312		1312	1312		1312	1312	
Dietary items												
Fruits, g/day	420 ± 259 *	305 ± 170 *	<0.001	404 ± 239 *	327 ± 183 *	<0.001	383 ± 238 *	370 ± 205 *	<0.001	444 ± 258 *	289 ± 172 *	<0.001
Vegetables, g/day	333 ± 149 *	310 ± 128 *	<0.001	331 ± 148 *	320 ± 132 *	0.001	319 ± 144 *	340 ± 138 *	<0.001	356 ± 154 *	292 ± 129 *	<0.001
Legumes, g/day	22 ± 13 *	19 ± 10 *	<0.001	22 ± 13 *	20 ± 10 *	<0.001	20 ± 12 *	21 ± 11 *	0.004	23 ± 13 *	19 ± 10 *	<0.001
Olive oil, g/day	27 ± 13 *	55 ± 14 *	<0.001	23 ± 10 *	57 ± 13 *	<0.001	36 ± 15 *	41 ± 19 *	<0.001	38 ± 16 *	43 ± 18 *	<0.001
Nuts, g/day	10 ± 12 *	22 ± 23 *	<0.001	10 ± 12 *	24 ± 24 *	<0.001	5 ± 6 *	34 ± 23 *	<0.001	17 ± 20 *	13 ± 15 *	<0.001
Total fish, g/day	97 ± 49 *	105 ± 47 *	<0.001	99 ± 50	103 ± 46 ^NS^	0.022	94 ± 46 *	107 ± 48 *	<0.001	104 ± 50 *	99 ± 47 *	0.017
Total cereals, g/day	203 ± 95 *	110 ± 52 *	<0.001	190 ± 94 *	118 ± 59 *	<0.001	196 ± 91 *	138 ± 69 *	<0.001	208 ± 93 *	118 ± 60 *	<0.001
Dairy products, g/day	402 ± 226 *	302 ± 177 *	<0.001	406 ± 220 *	292 ± 175 *	<0.001	418 ± 223 *	324 ± 197 *	<0.001	348 ± 226 *	267 ± 202 *	<0.001
Total meat, g/day	136 ± 57 *	156 ± 61 *	<0.001	140 ± 59 *	148 ± 58 *	<0.001	152 ± 59 *	146 ± 59 *	<0.001	130 ± 52 *	172 ± 62 *	<0.001
Cookies, g/day	30 ± 35 *	25 ± 27 *	<0.001	32 ± 35 *	23 ± 25 *	<0.001	36 ± 39 *	25 ± 27 *	<0.001	23 ± 27 *	35 ± 35 *	<0.001
Alcohol, g/day	15 ± 20 *	8 ± 11 *	<0.001	13 ± 18 *	9 ± 12 *	<0.001	16 ± 20 *	9 ± 13 *	<0.001	16 ± 20 *	9 ± 12 *	<0.001
17-item MedDiet Q, score	8.27 ± 2.62 *	8.56 ± 2.69 *	0.005	8.19 ± 2.67 *	8.88 ± 2.61 *	<0.001	7.68 ± 2.57 *	9.07 ± 2.73 *	<0.001	9.09 ± 2.67 *	7.65 ± 2.54 *	<0.001
	***Trans* FA**			**Linoleic acid**			**Linolenic acid**			**ω-3 FA**		
Participants, *n*	1312	1312		1312	1312		1312	1312		1312	1312	
Dietary items												
Fruits, g/day	379 ± 207 *	334 ± 198 *	<0.001	390 ± 239 *	365 ± 206 *	<0.001	383 ± 237 *	378 ± 216 *	<0.001	321 ± 209 *	392 ± 229 *	<0.001
Vegetables, g/day	332 ± 137 *	309 ± 134 *	<0.001	335 ± 150 *	330 ± 137 *	0.001	326 ± 143 *	347 ± 139 *	<0.001	269 ± 125 *	377 ± 145 *	<0.001
Legumes, g/day	20 ± 12	21 ± 11	0.730	21 ± 12	21 ± 11	0.061	22 ± 12 *	21 ± 11 *	<0.001	19 ± 11 *	23 ± 12 *	<0.001
Olive oil, g/day	39 ± 17	40 ± 18	0.154	35 ± 15 *	42 ± 19 *	<0.001	42 ± 17 *	39 ± 17 *	<0.001	37 ± 18 *	42 ± 17 *	<0.001
Nuts, g/day	15 ± 18	15 ± 17	0.293	5 ± 7 *	32 ± 24 *	<0.001	9 ± 12 *	33 ± 20 *	<0.001	11 ± 16 *	18 ± 19 *	<0.001
Total fish, g/day	100 ± 49	101 ± 49	0.243	105 ± 51 *	101 ± 47 *	0.002	102 ± 51 *	105 ± 46 *	<0.001	50 ± 23 *	162 ± 37 *	<0.001
Total cereals, g/day	135 ± 74 *	163 ± 80 *	<0.001	191 ± 90 *	138 ± 70 *	<0.001	213 ± 88 *	128 ± 68 *	<0.001	145 ± 81	152 ± 75	0.140
Dairy products, g/day	322 ± 213 *	388 ± 204 *	<0.001	411 ± 225 *	326 ± 196 *	<0.001	391 ± 222 *	329 ± 199 *	<0.001	353 ± 214	349 ± 202	0.082
Total meat, g/day	112 ± 44 *	170 ± 63 *	<0.001	156 ± 59 *	144 ± 58 *	<0.001	161 ± 61 *	141 ± 57 *	<0.001	128 ± 58 *	156 ± 60 *	<0.001
Cookies, g/day	16 ± 24 *	43 ± 38 *	<0.001	33 ± 38 *	27 ± 29 *	<0.001	41 ± 41 *	21 ± 25 *	<0.001	27 ± 32 *	24 ± 27 *	0.001
Alcohol, g/day	7 ± 12 *	13 ± 16 *	<0.001	16 ± 19 *	9 ± 12 *	<0.001	19 ± 21 *	7 ± 11 *	<0.001	10 ± 15 *	11 ± 14 *	0.030
17-item MedDiet Q, score	9.82 ± 2.49 *	7.50 ± 2.59 *	<0.001	7.97 ± 2.58 *	8.86 ± 2.76 *	<0.001	7.67 ± 2.62 *	9.49 ± 2.59 *	<0.001	7.64 ± 2.57 *	9.46 ± 2.59 *	<0.001

Abbreviations: FA, fatty acids; MedDiet Q, Mediterranean Diet Questionnaire; MUFAs, monounsaturated fatty acids; PUFAs, polyunsaturated fatty acids; SFAs, saturated fatty acids. All values are means ± SDs unless otherwise indicated. All quartiles were included in the analysis. Pearson’s chi-square test was used for categorical variables, and 1-factor ANOVA was used for continuous variables. * *p* < 0.05 for between-group changes, after adjustment for multiple comparisons with the Bonferroni method. ^NS^ No statistical significance after post hoc test.

**Table 4:** Association between total dietary fat and specific types of fat with the Metabolic Syndrome components (as dichotomous variables).

	Quintiles					*p* Value
	1	2	3	4	5	
**Total fat**						
High blood pressure	1.00 (ref.)	0.99 (0.73, 1.35)	0.81 (0.61, 1.10)	0.96 (0.71, 1.30)	0.94 (0.69, 1.27)	0.615
Hyperglycemia	1.00 (ref.)	0.94 (0.78, 1.12)	1.13 (0,94, 1.36)	**1.23 (1.02, 1.48)**	**1.55 (1.28, 1.88)**	<0.001
Hypertriglyceridemia	1.00 (ref.)	0.81 (0.69, 0.95)	0.88 (0.74, 1.03)	0.87 (0.74, 1.02)	0.90 (0.76, 1.06)	0.153
Low HDL-c	1.00 (ref.)	1.06 (0.90, 1.25)	1.19 (1.01, 1.40)	1.20 (1.02, 1.42)	1.14 (0.96, 1.34)	0.133
Abdominal obesity	1.00 (ref.)	1.19 (0.81, 1.75)	1.33 (0.90, 1.98)	1.60 (1.06, 2.43)	1.75 (1.15, 2.68)	0.065
**MUFAs**						
High blood pressure	1.00 (ref.)	1.17 (0.86, 1.59)	0.99 (0.73, 1.33)	0.87 (0.65, 1.17)	0.97 (0.72, 1.31)	0.429
Hyperglycemia	1.00 (ref.)	0.97 (0.81, 1.16)	0.98 (0.81, 1.17)	**1.26 (1.04, 1.52)**	**1.45 (1.19, 1.75)**	<0.001
Hypertriglyceridemia	1.00 (ref.)	0.93 (0.79, 1.09)	0.91 (0.78, 1.07)	0.99 (0.84, 1.16)	0.98 (0.83, 1.15)	0.741
Low HDL-c	1.00 (ref.)	1.07 (0.91, 1.26)	1.10 (0.94, 1.30)	1.18 (1.00, 1.38)	1.14 (0.97, 1.34)	0.359
Abdominal obesity	1.00 (ref.)	1.12 (0.76, 1.66)	1.08 (0.73, 1.60)	1.58 (1.04, 2.42)	1.54 (1.01, 3.34)	0.111
**PUFAs**						
High blood pressure	1.00 (ref.)	0.94 (0.69, 1.28)	0.89 (0.65, 1.20)	0.87 (0.64, 1.17)	0.93 (0.69, 1.27)	0.902
Hyperglycemia	1.00 (ref.)	1.02 (0.85, 1.23)	1.23 (1.02, 1.48)	1.23 (1.02, 1.49)	1.10 (0.91, 1.33)	0.084
Hypertriglyceridemia	1.00 (ref.)	1.10 (0.93, 1.29)	0.92 (0.78, 1.08)	1.12 (0.95, 1.33)	1.05 (0.89, 1.24)	0.105
Low HDL-c	1.00 (ref.)	0.96 (0.81, 1.13)	0.96 (0.82, 1.14)	1.11 (0.94, 1.31)	1.12 (0.95, 1.32)	0.160
Abdominal obesity	1.00 (ref.)	1.05 (0.70, 1.58)	1.13 (0.74, 1.71)	1.06 (0.71, 1.60)	1.31 (0.86, 2.00)	0.771
**SFAs**						
High blood pressure	1.00 (ref.)	0.86 (0.64, 1.15)	1.10 (0.81, 1.50)	1.08 (0.80, 1.47)	0.95 (0.70, 1.28)	0.432
Hyperglycemia	1.00 (ref.)	1.17 (0.97, 1.41)	**1.25 (1.04, 1.51)**	**1.35 (1.12, 1.63)**	**1.58 (1.31, 1.92)**	<0.001
Hypertriglyceridemia	1.00 (ref.)	0.96 (0.81, 1.13)	**0.83 (0.71, 0.98)**	**0.85 (0.72, 1.00)**	**0.74 (0.63, 0.87)**	0.003
Low HDL-c	1.00 (ref.)	1.12 (0.95, 1.32)	1.00 (0.84, 1.18)	1.12 (0.94, 1.32)	0.94 (0.79, 1.11)	0.137
Abdominal obesity	1.00 (ref.)	1.07 (0.72, 1.61)	1.40 (0.91, 2.16)	1.02 (0.68, 1.53)	1.33 (0.87, 2.02)	0.404
***Trans* FA**						
High blood pressure	1.00 (ref.)	0.97 (0.72, 1.30)	1.27 (0.92, 1.74)	0.87 (0.64, 1.18)	1.14 (0.81, 1.60)	0.114
Hyperglycemia	1.00 (ref.)	0.94 (0.78, 1.14)	1.17 (0.96, 1.43)	1.03 (0.84, 1.26)	1.12 (0.90, 1.38)	0.191
Hypertriglyceridemia	1.00 (ref.)	0.93 (0.79, 1.09)	0.93 (0.79, 1.10)	0.95 (0.80, 1.13)	0.81 (0.68, 0.98)	0.246
Low HDL-c	1.00 (ref.)	0.99 (0.84, 1.16)	1.02 (0.87, 1.21)	0.92 (0.78, 1.10)	0.89 (0.74, 1.07)	0.489
Abdominal obesity	1.00 (ref.)	1.68 (1.07, 2.64)	1.28 (0.84, 1.95)	1.34 (0.87, 2.07)	1.44 (0.91, 2.30)	0.256
**Linoleic acid**						
High blood pressure	1.00 (ref.)	0.98 (0.72, 1.34)	0.77 (0.57, 1.04)	0.93 (0.68, 1.26)	0.91 (0.67, 1.23)	0.423
Hyperglycemia	1.00 (ref.)	1.08 (0.91, 1.32)	1.17 (0.97, 1.41)	1.24 (1.03, 1.50)	1.08 (0.90, 1.30)	0.222
Hypertriglyceridemia	1.00 (ref.)	1.00 (0.85, 1.18)	1.05 (0.89, 1.24)	1.14 (0.97, 1.35)	1.12 (0.95, 1.32)	0.354
Low HDL-c	1.00 (ref.)	0.90 (0.76, 1.06)	1.08 (0.91, 1.27)	1.14 (0.97, 1.34)	**1.18 (1.00, 1.39)**	0.009
Abdominal obesity	1.00 (ref.)	1.14 (0.75, 1.73)	1.11 (0.74, 1.69)	1.12 (0.75, 1.69)	1.19 (0.79, 1.79)	0.939
**Linolenic acid**						
High blood pressure	1.00 (ref.)	0.83 (0.61, 1.13)	0.82 (0.59, 1.15)	0.99 (0.69, 1.40)	0.82 (0.59, 1.14)	0.488
Hyperglycemia	1.00 (ref.)	1.05 (0.86, 1.27)	1.17 (0.95, 1.44)	1.08 (0.87, 1.34)	1.03 (0.84, 1.26)	0.614
Hypertriglyceridemia	1.00 (ref.)	1.10 (0.92, 1.30)	1.12 (0.94, 1.35)	1.09 (0.91, 1.32)	1.05 (0.88, 1.26)	0.744
Low HDL-c	1.00 (ref.)	1.00 (0.84, 1.19)	0.97 (0.80, 1.16)	0.94 (0.78, 1.14)	1.04 (0.87, 1.25)	0.822
Abdominal obesity	1.00 (ref.)	1.54 (1.02, 2.33)	1.36 (0.87, 2.12)	1.20 (0.77, 1.89)	1.53 (0.98, 2.41)	0.219
**ω-3 FA**						
High blood pressure	1.00 (ref.)	0.97 (0.73, 1.30)	1.45 (1.06, 1.99)	0.93 (0.69, 1.25)	1.03 (0.76, 1.40)	0.048
Hyperglycemia	1.00 (ref.)	1.13 (0.94, 1.36)	1.02 (0.85, 1.23)	1.08 (0.89, 1.30)	**1.33 (1.09, 1.62)**	0.036
Hypertriglyceridemia	1.00 (ref.)	0.86 (0.73, 1.02)	0.97 (0.82, 1.14)	0.91 (0.77, 1.08)	**0.77 (0.65, 0.91)**	0.016
Low HDL-c	1.00 (ref.)	0.91 (0.77, 1.07)	0.86 (0.73, 1.01)	0.97 (0.82, 1.14)	0.84 (0.71, 1.00)	0.175
Abdominal obesity	1.00 (ref.)	1.22 (0.81, 1.86)	1.05 (0.70, 1.57)	0.93 (0.62, 1.40)	1.50 (0.95, 2.38)	0.237

Abbreviations: CI, confidence interval; HDL-c, high density lipoprotein cholesterol; OR, odds ratio; ref., reference. Values are expressed as *n* (%) and OR (95% CI). Logistic regression analysis comparing the presence of Metabolic Syndrome and its components (independent variables) between quintiles of total dietary fat and specific types (dependent variable). Logistic regression analysis after adjustment for sex, age (continuous variable), body mass index (continuous variable), smoking habit (categorized variable), education (categorized variable), energy intake (continuous variable), alcohol intake (continuous variable), adherence to the Mediterranean Diet (continuous variable) and physical activity (continuous variable, expressed as MET·min/week). ^2^Participants who not responded the physical activity questionnaires and participants reporting outliers for total physical activity expressed as MET·min/week (at 3 or more standard deviations from the mean) were excluded from the analysis (i.e., 154 men and 196 women).

## Abbreviations

ALA: α-linolenic acid; ANOVA: analysis of variance; BMI: body mass index; CHD: coronary heart disease; CI: confidence interval; CLA: conjugated linoleic acid; CVD: cardiovascular disease; DHA: docosahexaenoic acid; EPA: eicosapentaenoic acid; erMedDiet: energy-restricted traditional Mediterranean Diet; FFQ: food frequency questionnaire; FQI: fat quality index; HDL-c: high-density lipoprotein cholesterol; HOMA-IR: homeostasis model assessment-insulin resistance; HTN: hypertension; LA: linoleic acid; MedDiet: Mediterranean diet; MetS: Metabolic Syndrome; MUFA: monounsaturated fatty acids; OR: odds ratio; PREDIMED: PREvención con DIeta MEDiterránea; PUFA: polyunsaturated fatty acids; RAPA: Rapid Assessment of Physical Activity Questionnaires; SD: standard deviations; SFA: saturated fatty acids; T2DM: type 2 diabetes mellitus; TFA trans-fatty acids; TG: triglyceride; UFA: unsaturated fatty acids; WC: waist circumference; ω-3 FA: ω-3 fatty acid.
